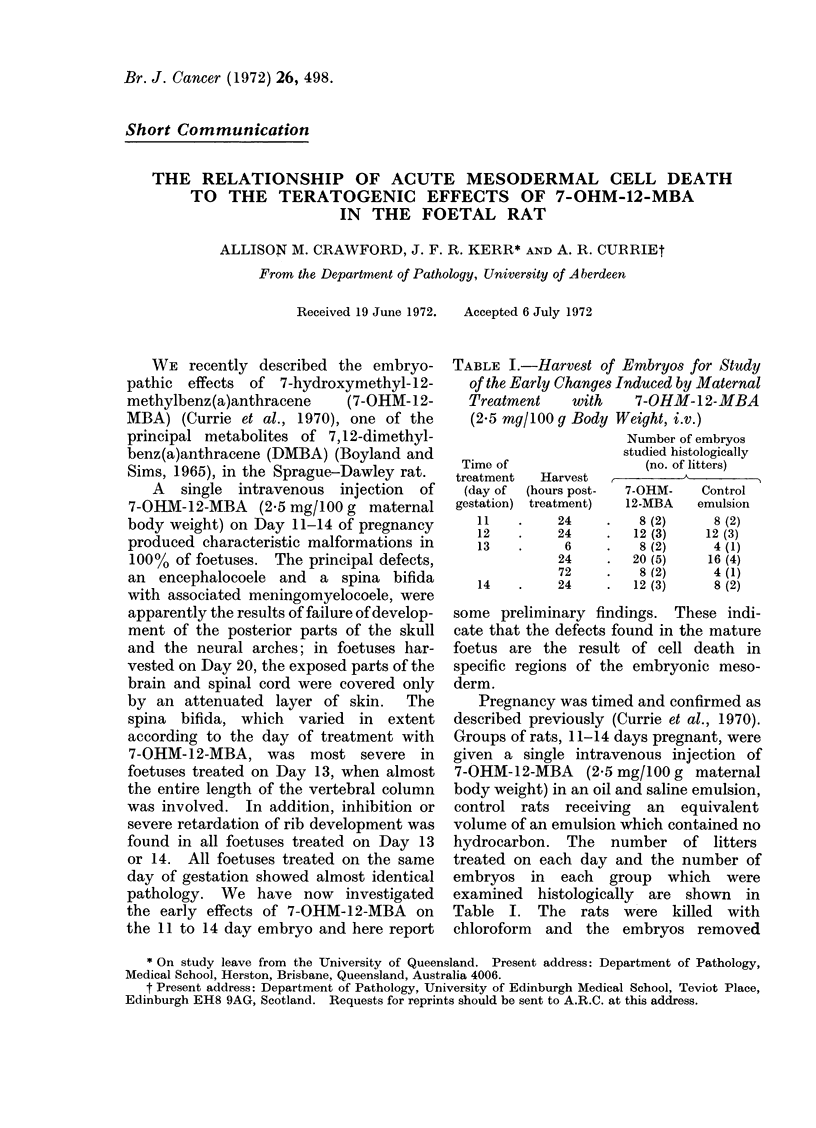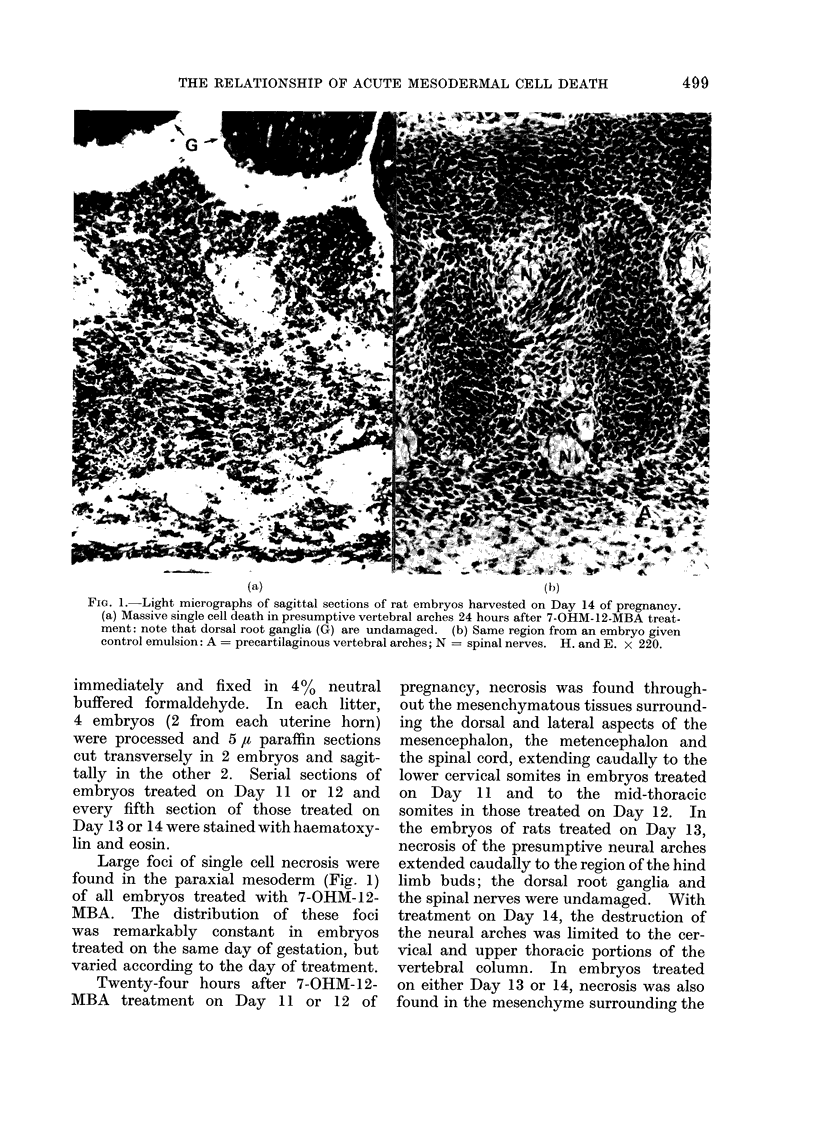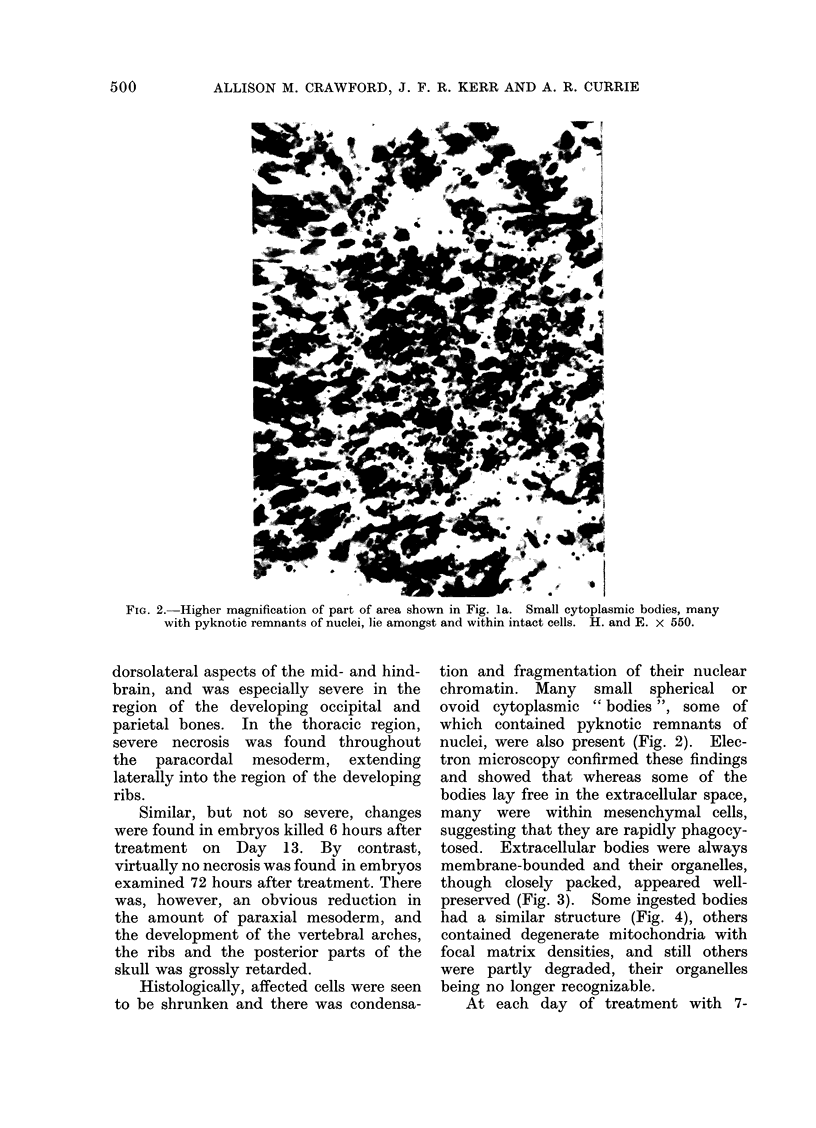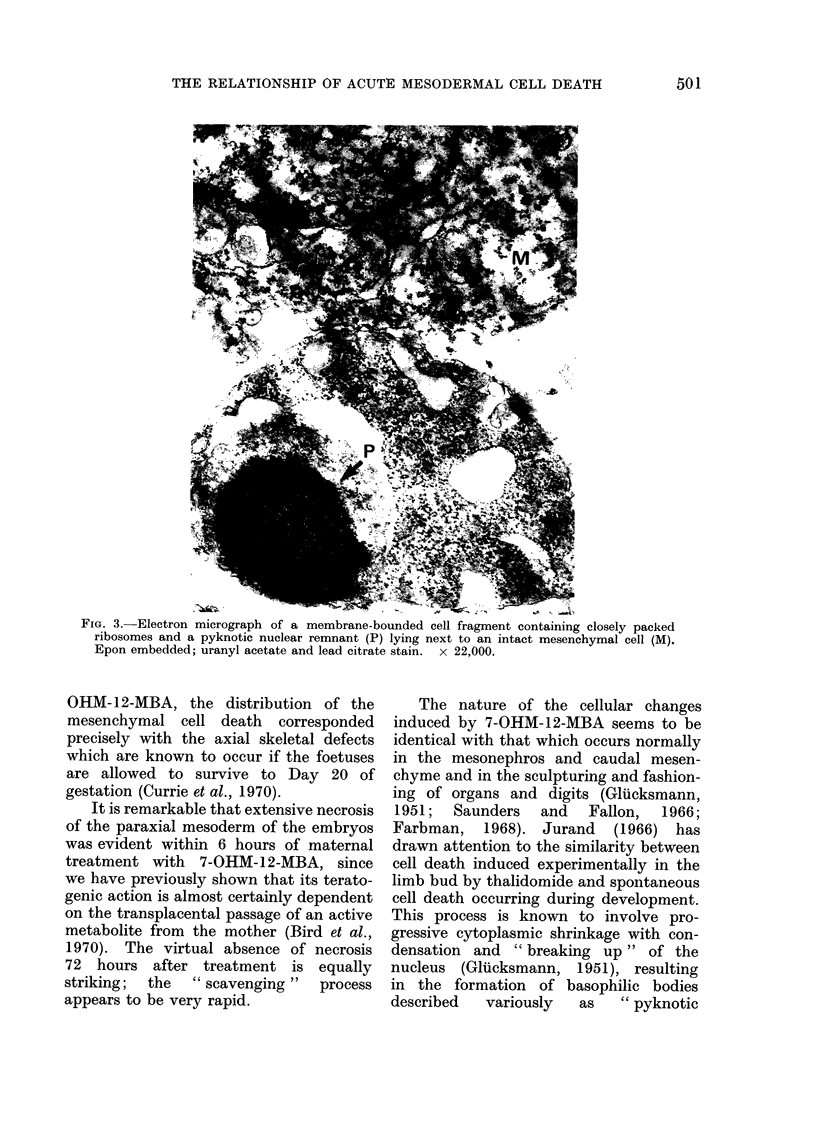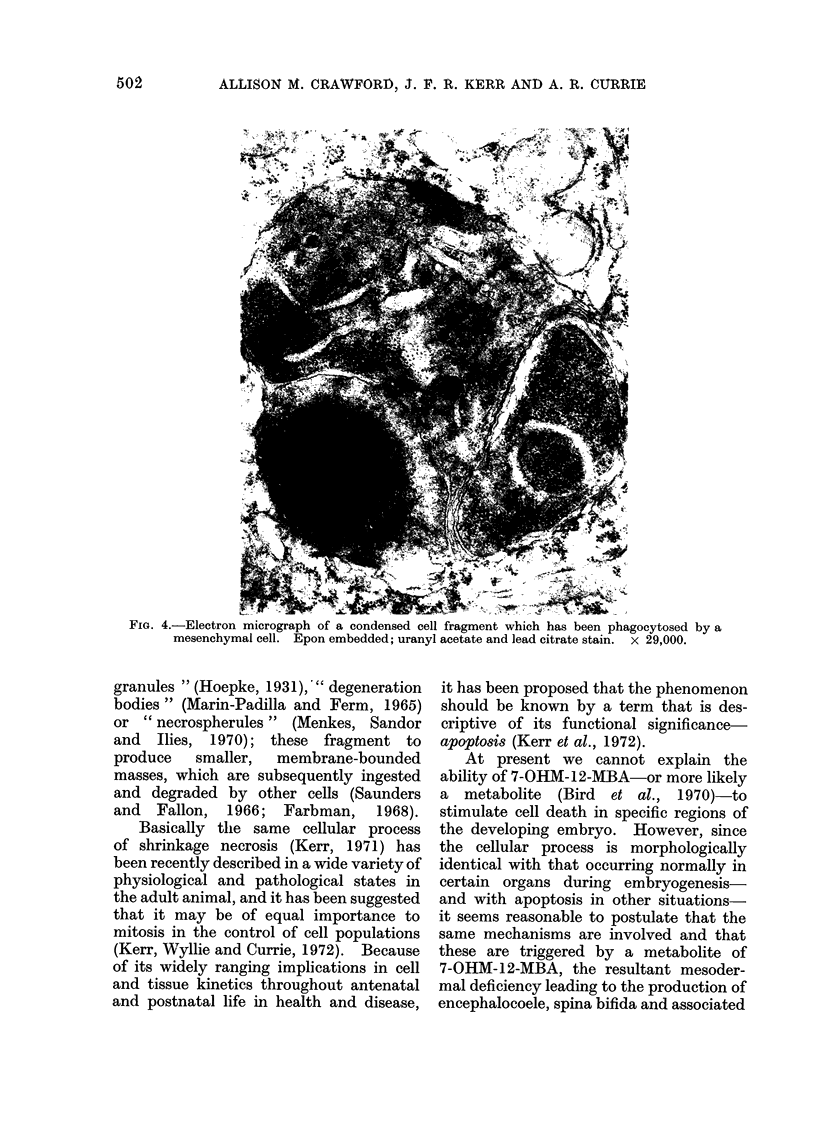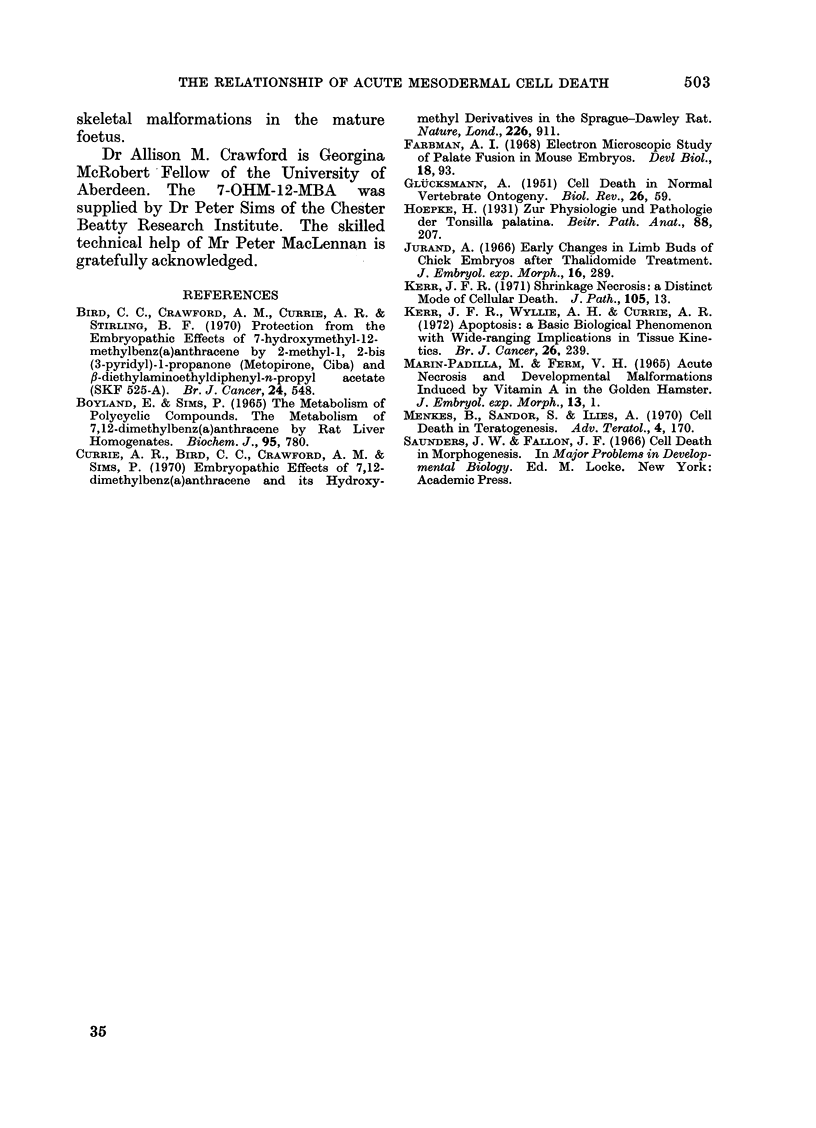# The relationship of acute mesodermal cell death to the teratogenic effects of 7-OHM-12-MBA in the foetal rat.

**DOI:** 10.1038/bjc.1972.67

**Published:** 1972-12

**Authors:** A. M. Crawford, J. F. Kerr, A. R. Currie

## Abstract

**Images:**


*Br. J. Cancer* (1972) **26**, 498.

*Short Communication*

### THE RELATIONSHIP OF ACUTE MESODERMAL CELL DEATH TO THE TERATOGENIC EFFECTS OF 7-OHM-12-MBA IN THE FOETAL RAT

ALLISON M. CRAWFORD, J. F. R. KERR* AND A. R. CURRIE†

*From the Department of Pathology, University of Aberdeen*

Received 19 June 1972. Accepted 6 July 1972

WE recently described the embryopathic effects of 7-hydroxymethyl-12-methylbenz(a)anthracene (7-OHM-12-MBA) (Currie *et al.*, 1970), one of the principal metabolites of 7,12-dimethylbenz(a)anthracene (DMBA) (Boyland and Sims, 1965), in the Sprague–Dawley rat.

A single intravenous injection of 7-OHM-12-MBA (2·5 mg/100 g maternal body weight) on Day 11–14 of pregnancy produced characteristic malformations in 100% of foetuses. The principal defects, an encephalocoele and a spina bifida with associated meningomyelocoele, were apparently the results of failure of development of the posterior parts of the skull and the neural arches; in foetuses harvested on Day 20, the exposed parts of the brain and spinal cord were covered only by an attenuated layer of skin. The spina bifida, which varied in extent according to the day of treatment with 7-OHM-12-MBA, was most severe in foetuses treated on Day 13, when almost the entire length of the vertebral column was involved. In addition, inhibition or severe retardation of rib development was found in all foetuses treated on Day 13 or 14. All foetuses treated on the same day of gestation showed almost identical pathology. We have now investigated the early effects of 7-OHM-12-MBA on the 11 to 14 day embryo and here report some preliminary findings. These indicate that the defects found in the mature foetus are the result of cell death in specific regions of the embryonic mesoderm.

TABLE I.—*Harvest of Embryos for Study of the Early Changes Induced by Maternal Treatment with 7-OHM-12-MBA (2·5 mg/100 g Body Weight, i.v.)*

| Time of treatment (day of gestation) | Harvest (hours post-treatment) | Number of embryos studied histologically (no. of litters): 7-OHM-12-MBA | Control emulsion |
|---|---|---|---|
| 11 | 24 | 8 (2) | 8 (2) |
| 12 | 24 | 12 (3) | 12 (3) |
| 13 | 6 | 8 (2) | 4 (1) |
| | 24 | 20 (5) | 16 (4) |
| | 72 | 8 (2) | 4 (1) |
| 14 | 24 | 12 (3) | 8 (2) |

Pregnancy was timed and confirmed as described previously (Currie *et al.*, 1970). Groups of rats, 11–14 days pregnant, were given a single intravenous injection of 7-OHM-12-MBA (2·5 mg/100 g maternal body weight) in an oil and saline emulsion, control rats receiving an equivalent volume of an emulsion which contained no hydrocarbon. The number of litters treated on each day and the number of embryos in each group which were examined histologically are shown in Table I. The rats were killed with chloroform and the embryos removed

* On study leave from the University of Queensland. Present address: Department of Pathology, Medical School, Herston, Brisbane, Queensland, Australia 4006.

† Present address: Department of Pathology, University of Edinburgh Medical School, Teviot Place, Edinburgh EH8 9AG, Scotland. Requests for reprints should be sent to A.R.C. at this address.

FIG. 1.—Light micrographs of sagittal sections of rat embryos harvested on Day 14 of pregnancy. (a) Massive single cell death in presumptive vertebral arches 24 hours after 7-OHM-12-MBA treatment: note that dorsal root ganglia (G) are undamaged. (b) Same region from an embryo given control emulsion: A = precartilaginous vertebral arches; N = spinal nerves. H. and E. × 220.

immediately and fixed in 4% neutral buffered formaldehyde. In each litter, 4 embryos (2 from each uterine horn) were processed and 5 $\mu$ paraffin sections cut transversely in 2 embryos and sagittally in the other 2. Serial sections of embryos treated on Day 11 or 12 and every fifth section of those treated on Day 13 or 14 were stained with haematoxylin and eosin.

Large foci of single cell necrosis were found in the paraxial mesoderm (Fig. 1) of all embryos treated with 7-OHM-12-MBA. The distribution of these foci was remarkably constant in embryos treated on the same day of gestation, but varied according to the day of treatment.

Twenty-four hours after 7-OHM-12-MBA treatment on Day 11 or 12 of pregnancy, necrosis was found throughout the mesenchymatous tissues surrounding the dorsal and lateral aspects of the mesencephalon, the metencephalon and the spinal cord, extending caudally to the lower cervical somites in embryos treated on Day 11 and to the mid-thoracic somites in those treated on Day 12. In the embryos of rats treated on Day 13, necrosis of the presumptive neural arches extended caudally to the region of the hind limb buds; the dorsal root ganglia and the spinal nerves were undamaged. With treatment on Day 14, the destruction of the neural arches was limited to the cervical and upper thoracic portions of the vertebral column. In embryos treated on either Day 13 or 14, necrosis was also found in the mesenchyme surrounding the

FIG. 2.—Higher magnification of part of area shown in Fig. 1a. Small cytoplasmic bodies, many with pyknotic remnants of nuclei, lie amongst and within intact cells. H. and E. × 550.

dorsolateral aspects of the mid- and hind-brain, and was especially severe in the region of the developing occipital and parietal bones. In the thoracic region, severe necrosis was found throughout the paracordal mesoderm, extending laterally into the region of the developing ribs.

Similar, but not so severe, changes were found in embryos killed 6 hours after treatment on Day 13. By contrast, virtually no necrosis was found in embryos examined 72 hours after treatment. There was, however, an obvious reduction in the amount of paraxial mesoderm, and the development of the vertebral arches, the ribs and the posterior parts of the skull was grossly retarded.

Histologically, affected cells were seen to be shrunken and there was condensation and fragmentation of their nuclear chromatin. Many small spherical or ovoid cytoplasmic "bodies", some of which contained pyknotic remnants of nuclei, were also present (Fig. 2). Electron microscopy confirmed these findings and showed that whereas some of the bodies lay free in the extracellular space, many were within mesenchymal cells, suggesting that they are rapidly phagocytosed. Extracellular bodies were always membrane-bounded and their organelles, though closely packed, appeared well-preserved (Fig. 3). Some ingested bodies had a similar structure (Fig. 4), others contained degenerate mitochondria with focal matrix densities, and still others were partly degraded, their organelles being no longer recognizable.

At each day of treatment with 7-

FIG. 3.—Electron micrograph of a membrane-bounded cell fragment containing closely packed ribosomes and a pyknotic nuclear remnant (P) lying next to an intact mesenchymal cell (M). Epon embedded; uranyl acetate and lead citrate stain. × 22,000.

OHM-12-MBA, the distribution of the mesenchymal cell death corresponded precisely with the axial skeletal defects which are known to occur if the foetuses are allowed to survive to Day 20 of gestation (Currie *et al.*, 1970).

It is remarkable that extensive necrosis of the paraxial mesoderm of the embryos was evident within 6 hours of maternal treatment with 7-OHM-12-MBA, since we have previously shown that its teratogenic action is almost certainly dependent on the transplacental passage of an active metabolite from the mother (Bird *et al.*, 1970). The virtual absence of necrosis 72 hours after treatment is equally striking; the "scavenging" process appears to be very rapid.

The nature of the cellular changes induced by 7-OHM-12-MBA seems to be identical with that which occurs normally in the mesonephros and caudal mesenchyme and in the sculpturing and fashioning of organs and digits (Glücksmann, 1951; Saunders and Fallon, 1966; Farbman, 1968). Jurand (1966) has drawn attention to the similarity between cell death induced experimentally in the limb bud by thalidomide and spontaneous cell death occurring during development. This process is known to involve progressive cytoplasmic shrinkage with condensation and "breaking up" of the nucleus (Glücksmann, 1951), resulting in the formation of basophilic bodies described variously as "pyknotic

FIG. 4.—Electron micrograph of a condensed cell fragment which has been phagocytosed by a mesenchymal cell. Epon embedded; uranyl acetate and lead citrate stain. × 29,000.

granules " (Hoepke, 1931), " degeneration bodies " (Marin-Padilla and Ferm, 1965) or " necrospherules " (Menkes, Sandor and Ilies, 1970); these fragment to produce smaller, membrane-bounded masses, which are subsequently ingested and degraded by other cells (Saunders and Fallon, 1966; Farbman, 1968).

Basically the same cellular process of shrinkage necrosis (Kerr, 1971) has been recently described in a wide variety of physiological and pathological states in the adult animal, and it has been suggested that it may be of equal importance to mitosis in the control of cell populations (Kerr, Wyllie and Currie, 1972). Because of its widely ranging implications in cell and tissue kinetics throughout antenatal and postnatal life in health and disease, it has been proposed that the phenomenon should be known by a term that is descriptive of its functional significance—*apoptosis* (Kerr *et al.*, 1972).

At present we cannot explain the ability of 7-OHM-12-MBA—or more likely a metabolite (Bird *et al.*, 1970)—to stimulate cell death in specific regions of the developing embryo. However, since the cellular process is morphologically identical with that occurring normally in certain organs during embryogenesis—and with apoptosis in other situations—it seems reasonable to postulate that the same mechanisms are involved and that these are triggered by a metabolite of 7-OHM-12-MBA, the resultant mesodermal deficiency leading to the production of encephalocoele, spina bifida and associated skeletal malformations in the mature foetus.

Dr Allison M. Crawford is Georgina McRobert Fellow of the University of Aberdeen. The 7-OHM-12-MBA was supplied by Dr Peter Sims of the Chester Beatty Research Institute. The skilled technical help of Mr Peter MacLennan is gratefully acknowledged.

### REFERENCES

Bird, C. C., Crawford, A. M., Currie, A. R. & Stirling, B. F. (1970) Protection from the Embryopathic Effects of 7-hydroxymethyl-12-methylbenz(a)anthracene by 2-methyl-1, 2-bis (3-pyridyl)-1-propanone (Metopirone, Ciba) and β-diethylaminoethyldiphenyl-*n*-propyl acetate (SKF 525-A). *Br. J. Cancer*, **24**, 548.

Boyland, E. & Sims, P. (1965) The Metabolism of Polycyclic Compounds. The Metabolism of 7,12-dimethylbenz(a)anthracene by Rat Liver Homogenates. *Biochem. J.*, **95**, 780.

Currie, A. R., Bird, C. C., Crawford, A. M. & Sims, P. (1970) Embryopathic Effects of 7,12-dimethylbenz(a)anthracene and its Hydroxymethyl Derivatives in the Sprague–Dawley Rat. *Nature, Lond.*, **226**, 911.

Farbman, A. I. (1968) Electron Microscopic Study of Palate Fusion in Mouse Embryos. *Devl Biol.*, **18**, 93.

Glücksmann, A. (1951) Cell Death in Normal Vertebrate Ontogeny. *Biol. Rev.*, **26**, 59.

Hoepke, H. (1931) Zur Physiologie und Pathologie der Tonsilla palatina. *Beitr. Path. Anat.*, **88**, 207.

Jurand, A. (1966) Early Changes in Limb Buds of Chick Embryos after Thalidomide Treatment. *J. Embryol. exp. Morph.*, **16**, 289.

Kerr, J. F. R. (1971) Shrinkage Necrosis: a Distinct Mode of Cellular Death. *J. Path.*, **105**, 13.

Kerr, J. F. R., Wyllie, A. H. & Currie, A. R. (1972) Apoptosis: a Basic Biological Phenomenon with Wide-ranging Implications in Tissue Kinetics. *Br. J. Cancer*, **26**, 239.

Marin-Padilla, M. & Ferm, V. H. (1965) Acute Necrosis and Developmental Malformations Induced by Vitamin A in the Golden Hamster. *J. Embryol. exp. Morph.*, **13**, 1.

Menkes, B., Sandor, S. & Ilies, A. (1970) Cell Death in Teratogenesis. *Adv. Teratol.*, **4**, 170.

Saunders, J. W. & Fallon, J. F. (1966) Cell Death in Morphogenesis. In *Major Problems in Developmental Biology*. Ed. M. Locke. New York: Academic Press.